# A bibliometric analysis of biopesticides in corn pest management: Current trends and future prospects

**DOI:** 10.1016/j.heliyon.2024.e40196

**Published:** 2024-11-06

**Authors:** Amik Krismawati, Yustisia Yustisia, Zainal Arifin, Titik Purbiati, Diding Rachmawati, Evy Latifah, Nicky Rahmana Putra, Irianto Irianto, Lailatul Qomariyah

**Affiliations:** a Research Center for Horticulture, National Research and Innovation Agency (BRIN), Complex Cibinong Science Center–BRIN, Cibinong, 16911, West Java, Indonesia; b Research Center for Food Crop, National Research and Innovation Agency (BRIN), Complex Cibinong Science Center–BRIN, Cibinong, 16911, West Java, Indonesia; c Research Center for Pharmaceutical Ingredients and Traditional Medicine, National Research and Innovation Agency (BRIN), Complex Cibinong Science Center–BRIN, Cibinong, 16911, West Java, Indonesia; d Department General Education, Faculty of Resilience, Rabdan Academy, Abu Dhabi, United Arab Emirates; e Department of Industrial Chemical Engineering, Institut Teknologi Sepuluh Nopember, 60111, Surabaya, Indonesia

**Keywords:** Bibliometric analysis, Biopesticides, Corn, Corn pest management, Corn production

## Abstract

This bibliographic review paper presents a comprehensive analysis of the scholarly literature on biopesticides utilized in corn pest management, employing a bibliometric approach to identify current trends and prospects in the field. The growing demand for sustainable agricultural practices has fueled interest in biopesticides as effective alternatives to conventional chemical pesticides. By systematically examining relevant publications, this review synthesizes the collective knowledge on biopesticide applications in corn production, encompassing various types of biopesticides, their modes of action, efficacy against key corn pests, and environmental considerations. The study synthesizes recent advances in microbial, botanical, and biochemical biopesticides such as Bacillus thuringiensis, neem extracts, and linalool, highlighting their specificity, minimal environmental impact, and potential to reduce pest resistance. It delves into the modes of action, including insecticidal activity, feeding disruption, and pest reproduction inhibition. The review also outlines an integrated pest management (IPM) strategy that combines biopesticides with agronomic practices, including crop rotation, biological control agents, and resistant crop varieties. This combined approach aims to enhance pest suppression, improve yield sustainability, and reduce chemical pesticide reliance. The findings provide valuable insights into sustainable corn pest management practices, promoting environmental conservation and agricultural productivity. Ultimately, this review aims to provide researchers, policymakers, and practitioners with a valuable resource for understanding the current landscape of biopesticides in corn pest management and guiding future research directions toward sustainable crop protection strategies.

## 1 Introduction

Global food demand has surged in recent decades, fueled by population growth and evolving dietary habits [1]. In response, agricultural practices have intensified to meet these rising demands, with corn cultivation playing a crucial role in ensuring nutritional diversity and supporting national economies [2]. However, the intensification of agriculture presents challenges, particularly in pest control [3]. While chemical pesticides have traditionally been used to safeguard crops, concerns over their environmental and health impacts have become increasingly prominent [4]. As a result, the search for eco-friendly alternatives has intensified, driven by the urgent need for sustainable food production solutions.

In modern agriculture, the sustainable management of pests and diseases poses a significant challenge, particularly in staple crops such as corn (*Zea mays* L.). Corn plays a crucial role in global food security, serving as a primary source of nutrition for humans and livestock and a vital feedstock for various industrial applications [5]. In 2022, the harvested area of corn amounted to an impressive 203,470,007 ha, contributing to a substantial production quantity of tons [6]. This vast expanse of cultivated land underscores the pivotal role of corn as a primary crop in global agriculture. Furthermore, the significant production output highlights the importance of corn as a staple food source for human consumption and a key component in livestock feed and industrial applications [7]. However, the prevalence of pests, including insects, pathogens, and weeds, threatens corn production, leading to substantial yield losses and economic impacts worldwide [[8], [9], [10]]. Traditional pest management strategies often rely heavily on synthetic pesticides, which, while effective in controlling pests, raise concerns regarding environmental pollution, human health risks, and the development of pesticide resistance among target organisms [[11], [12], [13]].

In response to these challenges, there has been a growing emphasis on exploring sustainable alternatives to synthetic pesticides, with biopesticides emerging as promising candidates for integrated pest management (IPM) in corn production systems [[14], [15], [16], [17]]. Biopesticides, derived from natural sources such as plants, microbes, and biological agents, offer several advantages over conventional pesticides, including reduced environmental impact, minimal residue levels, and target-specific modes of action [3,15,18]. These characteristics align with the principles of sustainable agriculture, promoting ecological balance, biodiversity conservation, and human health protection, as stated by Sustainable Development Goal (SDG) 2: “Zero Hunger” and SDG 15: “Life on Land".

The utilization of biopesticides in corn pest management represents a dynamic and evolving field of research, characterized by ongoing advancements in technology, formulation, and application strategies. He diversity of biopesticide options available for corn protection reflects the multifaceted nature of pest pressures and agronomic challenges farmers face across regions and cropping systems [19]. Microbial biopesticides, such as *Bacillus thuringiensis* (Bt) formulations, offer selective toxicity against specific insect pests, including corn earworm (*Helicoverpa zea*) and European corn borer (*Ostrinia nubilalis*), while botanical biopesticides, such as neem extracts and pyrethrum, provide alternative modes of action against a broader spectrum of pests [10,20].

To gain insights into the current status and prospects of biopesticides in corn pest management, this review employs a bibliometric analysis approach to systematically analyze the scholarly literature on this topic [[21], [22], [23], [24]]. Bibliometric analysis provides a quantitative framework for assessing publication trends, identifying research hotspots, mapping citation networks, and evaluating the impact of scientific contributions within a given field [[25], [26], [27], [28]]. By synthesizing the collective knowledge and research efforts in biopesticides for corn pest management, this review aims to provide valuable insights for researchers, policymakers, and practitioners seeking to enhance the sustainability and resilience of corn production systems.

## 2 Methodology

This study used bibliometric methods to analyze research on biopesticides for corn protection, going beyond typical analyses of individual authors and papers. By looking at journal citations, editorial input, and other metadata, it assessed the impact and value of publications and researchers in the field [29]. The SCOPUS database, known for its broad range of peer-reviewed studies, was chosen to gather the most relevant and recent articles for this topic [30]. A focused search string, (“Biopesticides”) AND (“Corn”), was crafted to ensure the studies found were closely related to our research goals.

Scopus was chosen as the database for several reasons. Firstly, Scopus is renowned for its comprehensive coverage of peer-reviewed literature across various disciplines, including agriculture [30]. It indexes various journals, conference proceedings, and other scholarly publications, ensuring access to a diverse and comprehensive collection of relevant articles for analysis. Additionally, Scopus provides access to many scholarly articles investigating biopesticides for corn protection. Its comprehensive coverage includes journals specializing in agriculture, plant sciences, environmental science, and related fields, making it well-suited for retrieving articles pertinent to the research topic.

Moreover, Scopus offers robust citation analysis features, allowing researchers to track citations, identify influential articles and authors, and explore citation networks within a specific research domain. This capability is valuable for conducting bibliometric analyses and evaluating the impact and visibility of scholarly publications. Finally, Scopus is widely recognized for its high-quality database and rigorous indexing standards, earning it a reputation for reliability and accuracy. This makes it a preferred choice for researchers seeking credible and trustworthy sources of scholarly information.

To capture the latest advancements, including studies published up to December 2023, only recent and English-language papers were included, resulting in a refined dataset of 170 documents. SCOPUS and VOSviewer software were then used for bibliometric mapping and visualization to uncover patterns within this dataset [32]. Following the methodological framework from Niknejad, Nazari [21], this approach enabled a systematic evaluation of the data, helping to identify key trends, leading authors, and emerging topics in biopesticide research for corn protection.

## 3 Data analysis and visualization

This study's data analysis and visualization section plays a pivotal role in uncovering insights, identifying patterns, and presenting findings derived from the collected data[31. In this section, we employ various analytical techniques and visualization tools to delve into the bibliometric data gathered from the selected articles on biopesticides for corn protection. Through rigorous analysis and visualization, we aim to elucidate trends, relationships, and critical characteristics within the scholarly literature, offering valuable insights into the research landscape in this domain.

Data analysis is the cornerstone of our investigation, enabling the collected information to be quantified and interpreted systematically. Statistical methods, trend analysis, and clustering techniques are applied to explore publication trends, citation patterns, author collaborations, and thematic concentrations within the dataset. Furthermore, through bibliometric mapping and network analysis, underlying structures and dynamics are uncovered, shedding light on the interconnectedness of research topics, influential authors, and emerging trends.

In parallel, visualization techniques are employed to enhance the interpretation and presentation of the findings. Visual representations, such as bibliographic networks, co-authorship maps, citation clusters, and keyword co-occurrence matrices, provide intuitive insights into the complex relationships and interactions within the literature. These visualizations facilitate the exploration of the data but also aid in effectively communicating the research outcomes to a diverse audience. Through a combination of rigorous data analysis and compelling visualization, a comprehensive understanding of the scholarly landscape surrounding biopesticides in corn pest management is offered in this section.

Table 1 provides an overview of bibliographic statistics for research on biopesticides for corn protection. A total of 170 documents were analyzed, spanning the period from 2000 to 2023. A diverse community of 801 authors contributed their expertise to these documents, producing an average of approximately 4.71 authors per document. The citation impact of the literature is notable, with an average of 19.87 citations per document and a cumulative total of 3379 citations across the dataset.

**Table 1:** Overview of bibliographic statistics for biopesticides for corn protection.

Description	Results
Documents	170
Period	2000:2023
Authors	801
Authors per document	4.71
Average citations per documents	19.87
Citations	3379
Document types	
Article	143
Review	10
Conference Paper	9
Book Chapter	8

The document types included in the analysis exhibit a variety of scholarly contributions. *Most documents* are articles, comprising 143 out of the total 170. The dataset includes ten reviews, 9 conference papers, and 8 book chapters. This distribution highlights the multifaceted nature of research on biopesticides for corn protection, with studies ranging from original research articles to comprehensive reviews and contributions from conferences and edited volumes.

Trend analysis of the bibliographic statistics reveals several noteworthy patterns over the examined period. The increasing number of documents indicates a growing interest in biopesticides for corn protection among researchers over time. Similarly, the upward trend in average citations per document suggests an increasing recognition and impact of the scholarly literature in this field. Additionally, the diversity of document types reflects the interdisciplinary nature of biopesticide research, incorporating contributions from various publication formats to advance knowledge and understanding of corn pest management strategies.

### 3.1 Yearly output of scholarly publications

The analysis of the yearly output of scholarly publications for biopesticides in corn protection is a fundamental component in understanding the dynamics and trends within the agricultural pest management research landscape. This section focuses on quantifying and examining the annual production of scholarly literature on biopesticides specifically applied in the protection of corn crops. Through the systematic evaluation of the yearly output of publications, insights can be gained into the progression of research efforts, emerging topics, and fluctuations in scholarly activity over time.

Understanding the yearly output of scholarly publications is valuable for assessing the growth and evolution of research in biopesticides for corn protection. By tracking the number of publications over successive years, patterns of research activity, including periods of expansion, stagnation, or decline, can be identified. Furthermore, the analysis of yearly output allows for the identification of trends, such as the emergence of new research topics, shifts in research focus, and responses to evolving challenges and priorities within the field of corn pest management.

This section presents an analysis of the yearly output of scholarly publications for biopesticides in corn protection, spanning a specified timeframe. By examining publication trends, insights into the temporal dynamics of research activity in this domain are elucidated, providing a comprehensive overview of the scholarly landscape and contributing to a deeper understanding of the advancements and challenges in developing and applying biopesticides for corn pest management.

**Fig. 1:**
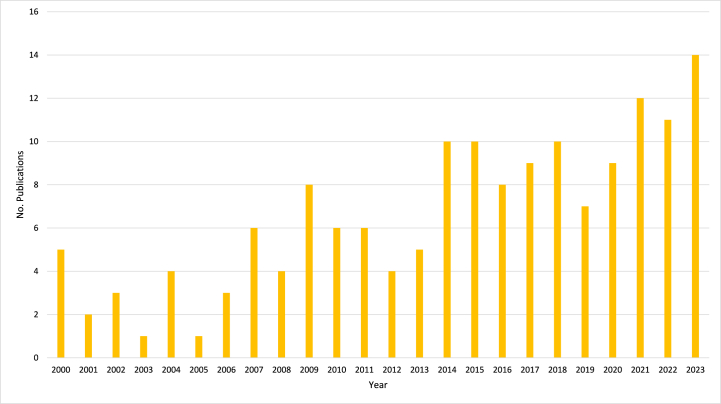
Annual publication output on Biopesticides in Corn Crop Protection.

Fig. 1 presents the annual publication output on biopesticides in corn crop protection from 2000 to 2023. The graph provides a visual representation of the number of scholarly publications released each year, offering insights into the temporal trends and dynamics of research activity within this area of agricultural science. A detailed examination of the graph reveals fluctuations in publication output over the 24 years, reflecting varying levels of scholarly interest and productivity in biopesticides for corn protection. For instance, there are peaks in publication output observed in specific years, such as 2015, 2018, and 2023, indicating periods of heightened research activity and productivity. Conversely, there are also troughs in publication count evident in years like 2005, 2013, and 2019, suggesting comparatively lower levels of scholarly output during those periods.

Overall, the trend depicted in Fig. 1 demonstrates a gradual increase in scholarly publications on biopesticides in corn crop protection over the examined period. While there are fluctuations from year to year, the general trajectory indicates a growing interest and investment in research related to biopesticide usage for corn pest management. This trend reflects the evolving landscape of agricultural science, with researchers and practitioners increasingly exploring alternative and sustainable pest management strategies, such as biopesticides, to address the challenges facing corn production. By visualizing the annual publication output, Fig. 1 provides valuable insights into the progression of research efforts, emerging trends, and focus areas within biopesticides for corn crop protection. This analysis enhances our understanding of the scholarly landscape and informs future research directions and priorities in sustainable pest management practices for corn production.

### 3.2 Geographical distribution of publications on biopesticides in corn crop protection

The geographical distribution of publications on biopesticides in corn crop protection is crucial in understanding the global research landscape and the regional focus of scholarly efforts in agricultural pest management. This section focuses on delving into the spatial distribution of scholarly publications, emphasizing examining the geographic regions where research on biopesticides for corn protection is concentrated. By analyzing the geographical distribution of publications, insights can be gleaned into the regional priorities, research collaborations, and disparities in research activity worldwide.

Understanding the geographical distribution of publications is valuable for gaining insights into the global dissemination of knowledge and the regional context of research on biopesticides for corn crop protection. By mapping the locations of affiliated institutions, author affiliations, and study sites reported in the publications, patterns of research activity can be identified, highlighting regions with significant contributions to the scholarly discourse on biopesticides in corn pest management.

This section analyzes the geographical distribution of publications on biopesticides in corn crop protection, examining the distribution of scholarly activity across different continents, countries, and regions. By exploring spatial patterns and trends, efforts are made to elucidate the global research landscape and provide insights into the geographic factors influencing research priorities and collaborations in biopesticides for corn crop protection.

**Fig. 2:**
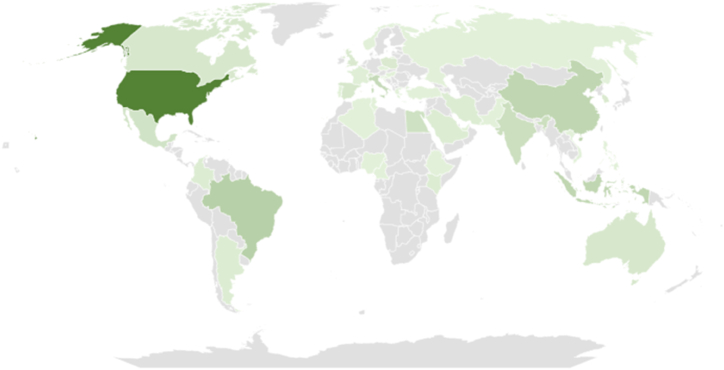
The dispersion of publications across regions related biopesticides in Corn Crop protection.

**Fig. 3:**
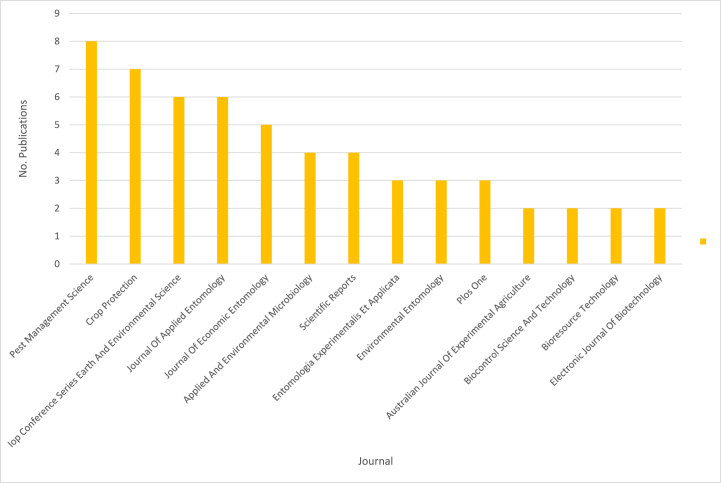
The top journals with the most citations related biopesticides for corn protection.

Fig. 2 visualizes the global distribution of research on biopesticides for corn protection, based on the affiliations of contributing authors. Key contributors include the United States, Brazil, and China, highlighting concentrated scholarly activity in these countries. Different shades of green represent varying productivity levels, with darker shades indicating higher research output, while blue indicates regions with no publications. This approach, centered on corresponding authors' locations, reveals a global landscape of research engagement, showing both the productivity and collaborative nature of efforts to advance knowledge in biopesticides for corn protection (see Fig. 3).

Table 2 presents the 20 most prolific nations ranked by the affiliations of corresponding authors related to biopesticides in corn crop protection. The table reveals the distribution of scholarly output across different countries, providing insights into the global research landscape in this field. The United States emerges as the leading contributor, with 52 articles authored by corresponding authors affiliated with institutions in the country. Following closely behind is Brazil, ranking second with 16 articles, highlighting the significant research activity in South America on biopesticides for corn pest management.

**Table 2:** The 20 most prolific nations ranked by the affiliations of corresponding authors related Biopesticides in Corn Crop Protection.

Rank	Country	Numbers of Article
1	United States	52
2	Brazil	16
3	China	13
4	Indonesia	13
5	Italy	11
6	India	9
7	Egypt	8
8	Mexico	7
9	Australia	5
10	Canada	5
11	Spain	5
12	Switzerland	5
13	Poland	4
14	South Korea	4
15	United Kingdom	4
16	Argentina	3
17	Colombia	3
18	Iran	3
19	Kenya	3
20	Saudi Arabia	3

China and Indonesia share the third position, with 13 articles each, underscoring the growing research interest and contributions from Asian countries in this domain. Italy and India also demonstrate substantial research output, ranking fifth and sixth, respectively, with 11 and 9 articles each. Egypt, Mexico, Australia, Canada, and Spain are among the top contributors, each producing a notable number of articles on biopesticides for corn crop protection.

Overall, Table 2 provides a comprehensive overview of the global distribution of scholarly publications on biopesticides in corn crop protection, highlighting the diverse geographical representation of research efforts in this area. The table underscores the collaborative nature of research, with contributions from nations spanning multiple continents. This analysis offers valuable insights into the regional priorities, research collaborations, and disparities in research activity across different countries, contributing to a deeper understanding of the global research landscape in biopesticides for corn pest management.

### 3.3 Identification of top-cited publications and the 20 most cited journals in the context of biopesticides in corn crop protection

In Section 4.3, focus is placed on the identification of top-cited publications and the 20 most cited journals in the context of biopesticides in corn crop protection. By analyzing citation data, the aim is to identify seminal works and authoritative sources that have significantly influenced research in this field. This analysis provides insights into the most impactful research contributions and the leading academic journals publishing research on biopesticides for corn pest management. Table 3 highlights the top 20 most cited publications on biopesticides for corn crop protection, providing an in-depth understanding of the most influential works in the field. The citations indicate the impact and importance of these research articles within the scientific community, showcasing how each has contributed to advancing biopesticide research. This table reveals the diversity of topics, including biological control, genetic modifications, microbial agents, and innovative biopesticide formulations, which are central to the ongoing evolution of sustainable pest management in corn agriculture.

**Table 3:** The 20 documents with the highest citation counts related Biopesticides in Corn Crop Protection.

Authors	Title	Year	Source title	Total Citation
Dorner J.W.	Biological control of aflatoxin contamination of crops	2004	Journal of Toxicology - Toxin Reviews	163
Li H.; Guan R.; Guo H.; Miao X.	New insights into an RNAi approach for plant defence against piercing-sucking and stem-borer insect pests	2015	Plant Cell and Environment	142
Ellis R.T.; Stockhoff B.A.; Stamp L.; Schnepf H.E.; Schwab G.E.; Knuth M.; Russell J.; Cardineau G.A.; Narva K.E.	Novel *Bacillus thuringiensis* binary insecticidal crystal proteins active on western corn rootworm, *Diabrotica virgifera* LeConte	2002	Applied and Environmental Microbiology	126
Weselowski B.; Nathoo N.; Eastman A.W.; MacDonald J.; Yuan Z.-C.	Isolation, identification and characterization of Paenibacillus polymyxa CR1 with potentials for biopesticide, biofertilization, biomass degradation and biofuel production	2016	BMC Microbiology	113
Kramer K.J.; Morgan T.D.; Throne J.E.; Dowell F.E.; Bailey M.; Howard J.A.	Transgenic avidin maize is resistant to storage insect pests	2000	Nature Biotechnology	102
Campos E.V.R.; Proença P.L.F.; Oliveira J.L.; Pereira A.E.S.; De Morais Ribeiro L.N.; Fernandes F.O.; Gonçalves K.C.; Polanczyk R.A.; Pasquoto-Stigliani T.; Lima R.; Melville C.C.; Della Vechia J.F.; Andrade D.J.; Fraceto L.F.	Carvacrol and linalool co-loaded in β-cyclodextrin-grafted chitosan nanoparticles as sustainable biopesticide aiming pest control	2018	Scientific Reports	89
Park Y.; Abdullah M.A.F.; Taylor M.D.; Rahman K.; Adang M.J.	Enhancement of Bacillus thuringiensis Cry3Aa and Cry3Bb toxicities to coleopteran larvae by a toxin-binding fragment of an insect cadherin	2009	Applied and Environmental Microbiology	88
Bateman M.L.; Day R.K.; Luke B.; Edgington S.; Kuhlmann U.; Cock M.J.W.	Assessment of potential biopesticide options for managing fall armyworm (*Spodoptera frugiperda*) in Africa	2018	Journal of Applied Entomology	84
Pascoli M.; Jacques M.T.; Agarrayua D.A.; Avila D.S.; Lima R.; Fraceto L.F.	Neem oil based nanopesticide as an environmentally-friendly formulation for applications in sustainable agriculture: An ecotoxicological perspective	2019	Science of the Total Environment	79
Amsellem Z.; Zidack N.K.; Quimby Jr. P.C.; Gressel J.	Long-term dry preservation of viable mycelia of two mycoherbicidal organisms	1999	Crop Protection	68
Tamez-Guerra P.; McGuire M.R.; Behle R.W.; Hamm J.J.; Sumner H.R.; Shasha B.S.	Sunlight persistence and rainfastness of spray-dried formulations of baculovirus isolated from *Anagrapha falcifera* (Lepidoptera: Noctuidae)	2000	Journal of Economic Entomology	63
Tamez-Guerra P.; Mcguire M.R.; Behle R.W.; Shasha B.S.; Galán Wong L.J.	Assessment of microencapsulated formulations for improved residual activity of Bacillus thuringiensis	2000	Journal of Economic Entomology	61
Kuate A.F.; Hanna R.; Doumtsop Fotio A.R.P.; Abang A.F.; Nanga S.N.; Ngatat S.; Tindo M.; Masso C.; Ndemah R.; Suh C.; Fiaboe K.K.M.	Spodoptera frugiperda Smith (Lepidoptera: Noctuidae) in Cameroon: Case study on its distribution, damage, pesticide use, genetic differentiation and host plants	2019	PLoS ONE	59
Devare M.; Londoño-R L.M.; Thies J.E.	Neither transgenic Bt maize (MON863) nor tefluthrin insecticide adversely affect soil microbial activity or biomass: A 3-year field analysis	2007	Soil Biology and Biochemistry	59
Crespo A.L.B.; Spencer T.A.; Alves A.P.; Hellmich R.L.; Blankenship E.E.; Magalhães L.C.; Siegfried B.D.	On-plant survival and inheritance of resistance to Cry1Ab toxin from Bacillus thuringiensis in a field-derived strain of European corn borer, Ostrinia nubilalis	2009	Pest Management Science	56
Alves A.P.; Lorenzen M.D.; Beeman R.W.; Foster J.E.; Siegfried B.D.	RNA interference as a method for target-site screening in the western corn rootworm, Diabrotica virgifera virgifera	2010	Journal of Insect Science	49
Douville M.; Gagné F.; Masson L.; McKay J.; Blaise C.	Tracking the source of Bacillus thuringiensis Cry1Ab endotoxin in the environment	2005	Biochemical Systematics and Ecology	49
Sparks M.E.; Shelby K.S.; Kuhar D.; Gundersen-Rindal D.E.	Transcriptome of the invasive brown marmorated stink bug, halyomorpha halys (sta° l) (heteroptera: Pentatomidae)	2014	PLoS ONE	43
Abouziena H.F.H.; Omar A.A.M.; Sharma S.D.; Singh M.	Efficacy comparison of some new natural-product herbicides for weed control at two growth stages	2009	Weed Technology	43
Zhou L.; Jiang H.-X.; Sun S.; Yang D.-D.; Jin K.-M.; Zhang W.; He Y.-W.	Biotechnological potential of a rhizosphere Pseudomonas aeruginosa strain producing phenazine-1-carboxylic acid and phenazine-1-carboxamide	2016	World Journal of Microbiology and Biotechnology	42

The most cited paper, by Dorner [32], is a cornerstone study with 163 citations. Published in the Journal of Toxicology - Toxin Reviews, this research delves into the biological control of aflatoxin contamination in crops, a critical issue in food safety. Aflatoxins are produced by fungi, notably Aspergillus species, which contaminate corn and other crops. Dorner's study emphasizes biocontrol methods using non-chemical agents to reduce aflatoxin contamination, offering safer alternatives to chemical fungicides. This work significantly impacts research on mycotoxin management and underscores the increasing reliance on biological methods for protecting food quality and public health.

The second most cited article, by Li, Guan [33], has 142 citations and introduces groundbreaking insights into RNA interference (RNAi) for plant defense. Published in Plant Cell and Environment, this research uses RNAi to defend plants against piercing-sucking and stem-boring insect pests, which are significant threats to corn. RNAi represents an advanced molecular technology where gene expression is silenced to protect plants from pests. This study showcases the potential of genetic methods in reducing dependency on chemical pesticides, offering a more targeted and environmentally friendly approach. The high citation count reflects the growing interest in RNAi as an innovative pest management tool.

Ellis, Stockhoff [34], with 126 citations, investigates novel *Bacillus thuringiensis*(Bt) insecticidal crystal proteins, which are active against the western corn rootworm, a notorious corn pest. Published in *Applied and Environmental Microbiology*, this work highlights the continuous development of Bt proteins to enhance pest control. The research is significant because it advances the understanding of microbial biopesticides and their role in combating resistant pest species. Bt-based products are widely used in genetically modified crops, and this paper strengthens the foundation of biopesticide research by expanding the range of effective biocontrol agents.

Another notable study is Weselowski, Nathoo [35], which has 113 citations. Published in *BMC Microbiology*, it focuses on the multifunctional bacterium *Paenibacillus polymyxa*, which shows potential as a biopesticide, biofertilizer, and biofuel production agent. This research explores how a single microbial strain can contribute to multiple facets of sustainable agriculture, such as enhancing soil fertility and producing energy while protecting crops from pests. The high citation count demonstrates the broad appeal of this study, particularly in its relevance to holistic agricultural systems that integrate biopesticides with other biotechnologies.

The work by Kramer, Morgan [36], cited 102 times, explores the use of transgenic avidin maize, which is resistant to storage insect pests. Published in Nature Biotechnology, this research presents an essential breakthrough in developing genetically modified organisms (GMOs) for pest management. By engineering maize to produce avidin, a toxic protein to certain pests, the study offers an innovative approach to post-harvest pest control. This work has far-reaching implications for food security, as it provides a method to reduce losses during storage, a crucial aspect of maintaining a stable food supply.

The study by Campos, Proença [37], published in *Scientific Reports* and cited 89 times, investigates the formulation of natural compounds, such as carvacrol and linalool, in chitosan nanoparticles to create sustainable biopesticides. This innovative approach enhances natural pesticides' efficacy and environmental sustainability, addressing key challenges such as stability and bioavailability. The study's focus on green chemistry and nanotechnology is reflected in its high citation count, as it resonates with the global movement towards reducing chemical pesticide use and mitigating environmental impact.

Park, Abdullah [38], with 88 citations, examines how the toxicity of Bacillus thuringiensis Cry proteins can be enhanced to target coleopteran larvae. Published in Applied and Environmental Microbiology, this research contributes to optimizing Bt proteins for use in biopesticides. The study's relevance lies in its potential to improve the control of pests resistant to traditional Bt strains, highlighting the ongoing need to innovate within microbial biopesticide formulations.

Bateman, Day [12], cited 84 times, addresses the management of *Spodoptera frugiperda*(fall armyworm) in Africa. This pest poses a significant threat to corn production, particularly in regions with limited chemical control options. Published in *the Journal of Applied Entomology*, this research evaluates potential biopesticide options for pest management. The study's high citation count indicates its importance in offering sustainable solutions to combat this invasive species, which has devastated African corn crops.

The study by Pascoli, Jacques [39], with 79 citations, focuses on neem oil-based nanopesticides and their applications in sustainable agriculture. Published in *Science of the Total Environment*, the research underscores the importance of eco-friendly formulations in pest management. Neem oil is a well-known botanical pesticide, and this study demonstrates how nanotechnology can enhance its efficacy and environmental compatibility, reflecting a growing trend in biopesticide research that prioritizes sustainability.

Amsellem, Zidack [40], cited 68 times, focuses on the long-term preservation of mycoherbicidal organisms, fungi that control weeds. Published in *Crop Protection*, this research significantly contributes to the practical use of fungal biocontrol agents in agriculture. One of the significant challenges in deploying mycoherbicides is maintaining the viability of these organisms over extended periods, which is crucial for their storage, transportation, and eventual application in the field. Amsellem's study addresses this by exploring methods to preserve the effectiveness of mycoherbicides, ensuring they remain potent after long-term storage. This aspect is essential for widespread adoption in agricultural settings, as it enables farmers to access reliable biocontrol products when needed without concerns over degradation or loss of efficacy. By improving the longevity of fungal agents, this research plays a crucial role in advancing mycoherbicide development and facilitating their practical use in sustainable weed management.

Tamez-Guerra, McGuire [41] appears twice on the list with two studies; each cited 63 and 61 times, published in *the Journal of Economic Entomology*. These studies investigate the stability and residual activity of *Bacillus thuringiensis* (Bt) formulations under real-world field conditions. The research focuses on practical challenges such as rain fastness (the ability of biopesticides to remain effective after rainfall) and sunlight persistence (the ability to withstand degradation due to UV exposure). Both factors are critical in determining the overall effectiveness of microbial biopesticides like Bt, vulnerable to environmental conditions that can degrade their potency. These studies offer valuable insights into how different formulations of Bt can be optimized to perform consistently in the field. By addressing these practical concerns, Tamez-Guerra's work contributes significantly to ensuring that biopesticides remain viable and effective in diverse agricultural environments, ultimately improving their reliability and adoption by farmers.

Fotso Kuate, Hanna [42], with 59 citations, presents a comprehensive case study on the distribution, damage, and management of *Spodoptera frugiperda* (fall armyworm) in Cameroon. Published in *PLoS ONE*, this research is vital for its region-specific focus, providing detailed insights into the impact of the invasive fall armyworm on agriculture in sub-Saharan Africa. The fall armyworm has caused significant crop damage, particularly to maize, and has posed challenges for pest management in regions where biopesticide use is still developing. Kuate's study highlights the importance of local context in biopesticide research, showing how regional factors such as climate, pest behavior, and available resources affect the success of biopesticide strategies. The research underscores the need for tailored biopesticide solutions and localized management strategies by focusing on a region heavily impacted by this invasive species. The findings emphasize the critical role of invasive species management and the importance of adapting biopesticide use to the unique agricultural challenges of different geographic areas.

The study by Devare, Londoño-R [43], cited 59 times and published in Soil Biology and Biochemistry, investigates the ecological effects of genetically modified Bt maize on soil microbial activity. This long-term field study is pivotal in addressing concerns about the environmental sustainability of transgenic crops, especially Bt maize, which produces insecticidal proteins derived from Bacillus thuringiensis. Focusing on soil health and microbial communities, the research contributes to an ongoing debate about the unintended consequences of genetically modified crops on non-target organisms and ecosystem services. The findings are essential for evaluating the broader ecological impacts of Bt crops, including their potential to alter soil microbial diversity and function, which are crucial for maintaining soil fertility and overall ecosystem health.

The paper by Crespo, Spencer [44], published in Pest Management Science and cited 56 times, addresses the growing issue of resistance development in pests, particularly the European corn borer, to Bt toxins. This research is crucial for understanding how pests adapt to Bt-based biopesticides, which have been widely used in transgenic crops to control corn pests. The study highlights the need for integrated pest management strategies that combine Bt crops with other control methods to delay resistance development. The results underscore the importance of monitoring pest populations for resistance and implementing resistance management practices to maintain the long-term effectiveness of Bt crops in sustainable agriculture.

Alves, Lorenzen [45], with 49 citations and published in the Journal of Insect Science, explores RNA interference (RNAi) as a novel tool for pest management, particularly in targeting the western corn rootworm, a major pest of corn. RNAi is a gene-silencing technique that can disrupt specific genes in pests, leading to reduced pest populations without harming non-target organisms. This study contributes to the growing literature on RNAi-based biopesticides, offering a promising alternative to chemical pesticides and Bt toxins. The research provides insights into the genetic mechanisms that can be exploited to control pest populations more sustainably, marking a significant advancement in developing targeted biopesticides.

Douville, Gagné [46], also cited 49 times, focuses on the environmental persistence of Bacillus thuringiensis (Bt) endotoxins, which are widely used in Bt crops. Published in Biochemical Systematics and Ecology, this research is critical for understanding the long-term ecological impacts of Bt crops, particularly the accumulation and persistence of Bt toxins in the environment. The study examines how these toxins affect non-target organisms and ecosystems, addressing concerns about their potential to disrupt ecological balance. This research is foundational for risk assessments related to the widespread use of Bt crops and informs regulatory decisions on their environmental safety.

The study by Sparks, Shelby [47], with 43 citations and published in PLoS ONE, provides a transcriptome analysis of the brown marmorated stink bug, an invasive species that poses a significant threat to crops, including corn. This research offers valuable insights into the genetic basis of the stink bug's behavior and biology, which is essential for developing targeted biopesticide strategies. By identifying genes involved in the pest's feeding and reproductive processes, the study contributes to developing more effective control measures that target the stink bug without affecting beneficial insects or other non-target species.

Abouziena, Omar [48], with 43 citations, compares the efficacy of natural-product herbicides for weed control. Published in Weed Technology, this research supports the increasing use of natural herbicides as alternatives to synthetic chemicals, contributing to the broader movement toward sustainable agriculture. The study evaluates the effectiveness of these natural products in controlling weeds at different growth stages, offering practical insights for farmers seeking eco-friendly weed management solutions. This work is part of the growing trend toward reducing reliance on synthetic herbicides, which can have harmful environmental effects, and promoting safer, natural alternatives.

Lastly, Zhou, Jiang [49], with 42 citations and published in the World Journal of Microbiology and Biotechnology, explores the biotechnological potential of Pseudomonas aeruginosa, a rhizosphere bacterium known for producing bioactive compounds. This study focuses on producing phenazine-1-carboxylic acid and phenazine-1-carboxamide, compounds with pesticide properties. The research highlights the potential of microbial metabolites for use in biopesticide formulations, offering a sustainable solution for pest control. By harnessing the natural properties of microbes, this study contributes to developing eco-friendly pest management strategies, emphasizing the role of biotechnology in sustainable agriculture.

### 3.4 Author co-citation analysis in the context of biopesticides for corn protection

This section employs author co-citation analysis to explore the intellectual structure and scholarly networks within the research field of biopesticides for corn protection. The aim is to uncover critical researchers, influential works, and thematic clusters that drive research in this domain. By examining patterns of co-citation, pivotal authors and seminal papers that have significantly contributed to advancing knowledge and innovation in biopesticides for corn crop protection can be identified.

Author co-citation analysis provides valuable insights into researchers' interconnectedness and contributions to the scholarly discourse on biopesticides for corn protection. Collaborative networks and intellectual communities shaping research in this field are discerned by identifying authors who are frequently cited together in the literature. Additionally, co-citation analysis enables the identification of emerging trends, interdisciplinary collaborations, and gaps in the literature, thereby offering a comprehensive understanding of the research landscape and informing future research directions.

This section presents an in-depth analysis of the author's co-citation networks in the context of biopesticides for corn protection. Through the exploration of co-citation patterns, the underlying structure of the research field is revealed, influential authors and seminal works are identified, and the dynamics of knowledge dissemination and collaboration are elucidated. By employing advanced bibliometric techniques, valuable insights into the intellectual foundations and scholarly networks driving research in biopesticides for corn crop protection are provided, ultimately contributing to the advancement of sustainable pest management practices in agriculture.

**Fig. 4:**
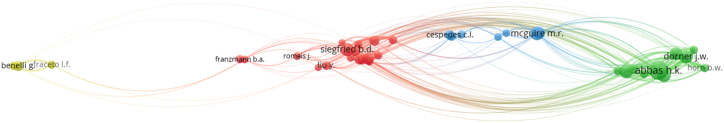
Minimum citation threshold of 20 and 348 link.

Fig. 4 visualizes authors' co-citations within biopesticide research for corn protection, highlighting collaborative networks and key figures in the field. Each color represents a distinct cluster of authors, with connecting lines illustrating relationships among clusters. Bubble sizes correspond to citation counts, emphasizing the impact of authors, with Abbas and Sigrieg standing out due to high citation numbers. A minimum citation threshold of 20 is applied, revealing 348 links, which represent co-citation connections among influential authors. This visualization captures the scholarly network, showcasing significant collaborations and shared references in biopesticide research for corn protection.

### 3.5 Co-citation of journals in the context of biopesticides for corn protection

In this section, the co-citation analysis of journals in the context of biopesticides for corn protection is conducted to uncover the intellectual structure and scholarly networks within this research field. Collaborative co-citation analysis provides valuable insights into the relationships between academic publications, influential journals, and thematic clusters driving research in biopesticides for corn crop protection. By examining patterns of co-citation, key journals, prominent themes, and emerging trends in the literature are identified, offering a nuanced understanding of the scholarly landscape and informing future research directions.

The co-citation analysis of journals offers a unique perspective on disseminating scholarly knowledge and the interdisciplinary nature of research in biopesticides for corn protection. Collaborative networks and intellectual communities shaping research in this field are discerned by identifying journals frequently co-cited together in the literature. Additionally, core journals that serve as hubs of knowledge dissemination and facilitate interdisciplinary dialogue among researchers from diverse backgrounds can be identified through co-citation analysis.

This section presents an in-depth analysis of journal co-citation networks in the context of biopesticides for corn protection. Through the exploration of co-citation patterns, the underlying structure of the research field is revealed, influential journals and thematic clusters are identified, and the dynamics of knowledge dissemination and collaboration within the scholarly community are elucidated. By employing advanced bibliometric techniques, valuable insights into the intellectual foundations and scholarly networks driving research in biopesticides for corn crop protection are provided, ultimately contributing to the advancement of sustainable pest management practices in agriculture.

**Fig. 5:**
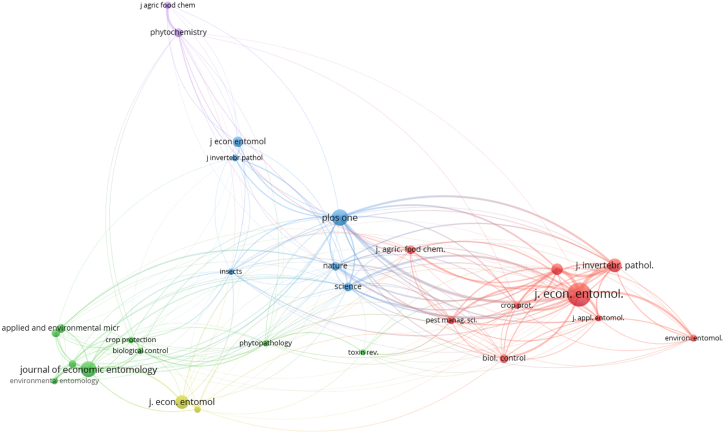
Co-citation of journals: minimum citation threshold of 20 and 201 links.

Examining the co-citation network among journals in biopesticides for corn protection is crucial for understanding this field's complex interconnections and thematic orientations. Fig. 5 provides insight into this network by establishing a minimum citation threshold of 20 to highlight journals with significant influence. With 201 links uncovered, this visualization illuminates the collaborative relationships and shared scholarly impact among journals. The deliberate choice of a minimum citation threshold ensures a focused analysis, prioritizing journals with notable contributions and influence shaping the discourse on biopesticides for crop protection. The prominence of journals such as the Journal of Economic Entomology in the co-citation network underscores their pivotal roles, justifying their central position and widespread influence in scholarly discussions concerning biopesticides for safeguarding corn crops.

### 3.6 Co-citation of references in the context of biopesticides for corn protection

In this section, the co-citation analysis of references in the context of biopesticides for corn protection is undertaken to unravel the underlying scholarly connections and thematic associations within this research domain. Through co-citation analysis of references, valuable insights into the literature's citation patterns and intellectual structure are provided, highlighting seminal works, influential research themes, and emerging trends in biopesticides for corn crop protection. By examining patterns of co-citation among references, key research clusters, pivotal studies, and influential authors can be identified, offering a comprehensive understanding of the scholarly landscape and informing future research directions.

The co-citation analysis of references offers a unique perspective on disseminating knowledge and developing research themes in biopesticides for corn protection. Clusters of related research and seminal works that have significantly impacted the field can be discerned by identifying references frequently co-cited in the literature. Additionally, research trends and literature gaps can be identified through co-citation analysis, providing valuable insights for researchers and practitioners seeking to advance knowledge and innovation in sustainable pest management practices for corn crops.

This section presents an in-depth analysis of reference co-citation networks in the context of biopesticides for corn protection. Through the exploration of co-citation patterns, the field's intellectual foundations are uncovered, key research clusters and influential studies are identified, and the dynamics of knowledge dissemination and collaboration within the scholarly community are elucidated. By employing advanced bibliometric techniques, valuable insights into the scholarly networks driving research in biopesticides for corn crop protection are provided, ultimately contributing to advancing sustainable agriculture practices and mitigating environmental impacts associated with conventional pesticide use in corn production.

**Fig. 6:**
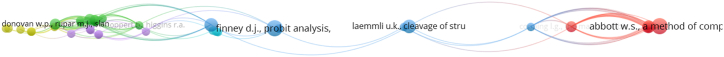
Co-citation of references: threshold of 2 and 240 link.

Fig. 6 offers an in-depth visualization of bibliographic coupling among articles in biopesticides for corn protection, with clusters marked by distinct colors. Each bubble's size reflects the article's total citations, enabling a quick visual grasp of citation impact, where larger bubbles signify higher citation counts. The colors differentiate clusters of thematically or conceptually linked articles based on shared bibliographic references, while the spatial layout illustrates articles' bibliographic relationships; articles positioned closely share numerous references, underscoring their thematic interconnectedness. In this network, spatial proximity between articles signifies a strong overlap in cited references, indicating significant bibliographic connections. Through the combination of bubble size and spatial arrangement, this visualization offers an intuitive portrayal of the articles' interconnectedness within the field, allowing for a clear understanding of the major influential works and their impact within biopesticides for corn protection research.

Additionally, Fig. 6 applies a minimum citation threshold of 2, uncovering 240 links that highlight significant bibliographic overlaps among articles. The size of each bubble underscores the article's citation impact, while color-coded clusters represent collections of articles with substantial thematic links. With this threshold, the visualization emphasizes impactful articles and provides a focused representation of bibliographic coupling among frequently cited works, showcasing the cohesive network within this research area. The 240 links reveal an interconnected literature landscape, offering insights into key themes and influential articles shaping biopesticide research for corn protection.

### 3.7 Bibliographic coupling of countries in the context of biopesticides for corn protection

This section explores the bibliographic coupling of countries in the context of biopesticides for corn protection, aiming to uncover collaborative networks and international research partnerships within this field. Valuable insights into the extent of collaboration and knowledge exchange among different nations are provided by the bibliographic coupling of countries, highlighting key players and regional dynamics in biopesticide research for corn crop protection by patterns of bibliographic coupling, countries that frequently collaborate on research related to biopesticides for corn can be identified, shedding light on global research trends and collaborative efforts aimed at addressing challenges in pest management and sustainable agriculture.

The bibliographic coupling of countries offers a unique perspective on the international landscape of research in biopesticides for corn protection. Countries that frequently co-publish articles on this topic can be identified, discerning patterns of collaboration and cooperation, as well as regional disparities in research output and impact. Additionally, the influence of different countries in shaping the discourse on biopesticides for corn protection can be assessed through bibliographic coupling, highlighting leading contributors and emerging research hubs worldwide.

This section presents an in-depth analysis of bibliographic coupling among countries in the context of biopesticides for corn protection. Through exploring bibliographic coupling patterns, collaborative networks are uncovered, influential countries are identified, and the dynamics of international research partnerships in this field are elucidated. By employing advanced bibliometric techniques, valuable insights into the global landscape of research in biopesticides for corn crop protection are provided, ultimately contributing to the advancement of sustainable pest management practices and the promotion of international cooperation in agricultural research and innovation.

**Fig. 7:**
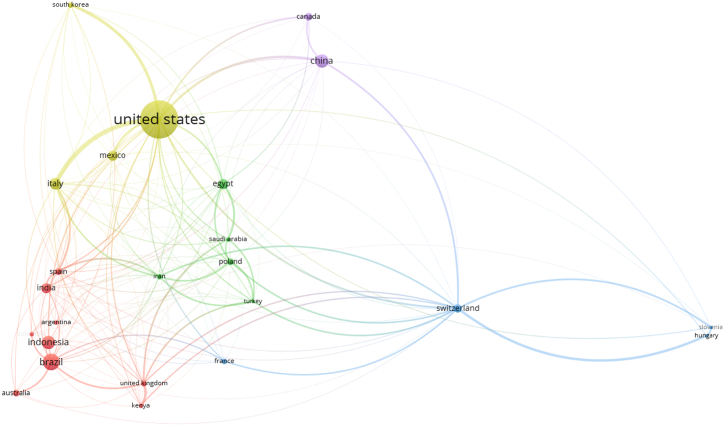
Bibliographic coupling of countries: minimum publication threshold of 2 documents and 261 links.

Fig. 7 displays the bibliographic coupling of countries engaged in biopesticide research for corn protection, with each bubble representing a country and its size reflecting the number of publications it contributes. The figure highlights the United States as a leading contributor, consistent with insights from Fig. 2 and Table 2. The varied bubble sizes provide a quick visual overview of each country's research output, underscoring their influence in this global research area. This visualization emphasizes the international scale of biopesticide research for corn protection, spotlighting certain countries' roles in advancing the field. Furthermore, by analyzing keyword co-occurrences, the VOSviewer software reveals thematic connections among countries, shedding light on collaborative and shared research interests worldwide.

The United States’ focus on biopesticides for corn protection is part of a broader global shift toward sustainable agriculture. This trend reflects growing awareness of the environmental costs of conventional pesticides and the need for eco-friendly pest management alternatives [50]. Biopesticides derived from natural sources support Sustainable Development Goal (SDG) 15: Life on Land, by promoting practices that reduce ecological risks and support biodiversity. By integrating environmentally sustainable methods, these countries contribute to conserving terrestrial ecosystems and aligning with international sustainability objectives [18].

Additionally, rising consumer demand for safe and healthy produce has significantly influenced the move toward biopesticides, supporting SDG 3: Good Health and Well-being. As consumers increasingly prioritize food safety, there is a growing market for corn cultivated through eco-friendly methods, furthering SDG 12: Responsible Consumption and Production. This trend has pushed agricultural practices to prioritize environmental and health considerations, contributing to goals such as SDG 2: Zero Hunger. Moreover, supportive regulatory frameworks and government policies in these countries have bolstered biopesticide use, recognizing the dual benefits for the environment and public health. With ongoing investment in research and innovation, biopesticides have become essential for sustainable corn protection, addressing both export standards and biodiversity conservation needs within the United States and beyond.

### 3.8 Co-occurrence of authors’ keywords in the context of biopesticides for corn protection

Section 4.8 delves into the analysis of the co-occurrence of authors' keywords in the context of biopesticides for corn protection. This type of analysis helps identify recurring research themes, trends, and connections between different areas of study within the field. By setting a minimum keyword threshold of two, the study mapped out 236 keyword links, which provide insights into how frequently different concepts are associated. This approach highlights the relationships between various research focuses on biopesticides and corn protection, offering a clear picture of the dominant themes and emerging trends in this body of literature.

The co-occurrence map is a visual representation of these interconnected keywords. At the center of this network are the keywords “biopesticides” and “biological control,” which are the most frequently used terms and thus represented by the most significant nodes. Their central location indicates that much of the research in this field revolves around these core concepts, with a significant focus on utilizing biological agents and natural substances to control pests in corn production. These keywords are connected to prominent topics, such as “Bacillus thuringiensis” (Bt), a well-known microbial biopesticide. This indicates the widespread use of Bt-based products in corn pest management. Additionally, the term “Spodoptera frugiperda,” which refers to the fall armyworm, a significant pest in corn production, is closely linked to these core concepts. This reflects the critical importance of developing biopesticides targeted explicitly at managing this destructive pest.

Further analysis of the keyword clusters reveals additional essential themes. For example, keywords like “Beauveria bassiana” and “entomopathogenic fungi” indicate a strong focus on research into fungal biocontrol agents. Beauveria bassiana is a widely studied entomopathogenic fungus used to control various corn pests, and its close association with “biopesticides” and “biological control” suggests that fungal biocontrol agents are a significant research area in corn pest management. Moreover, the keyword “formulation” appears frequently on the map, emphasizing the importance of developing stable and effective biopesticide formulations that can perform reliably in diverse environmental conditions. Keywords like “rainfastness” point to specific formulation challenges that researchers are addressing, such as ensuring that biopesticides remain effective even after exposure to rain or sunlight.

The cluster containing keywords related to mycotoxins, such as “aflatoxin” and “aspergillus flavus,” highlights another critical research area: the use of biopesticides to control fungal pathogens that can contaminate corn with harmful toxins. This is particularly important for food safety, as mycotoxins like aflatoxins pose significant health risks when they contaminate food supplies. Research in this area focuses on how biopesticides can help prevent fungal infections and reduce the levels of these dangerous toxins in corn, thereby improving both crop quality and consumer safety.

Another exciting cluster centers on the development of transgenic plants and bioinsecticides, as evidenced by keywords like “transgenic plants,” “bioassays,” and “Bt maize.” The term “Bt maize” refers to genetically modified corn that expresses *Bacillus thuringiensis* toxins, making it resistant to certain insect pests. The close association of these terms with “biopesticides” and “biological control” indicates the integration of transgenic approaches into biopesticide research. This suggests that while traditional biocontrol methods are essential, genetic modifications that enhance pest resistance in crops are also a significant area of study.

Finally, the overall interconnectedness of the keywords shown in the map reveals a comprehensive and interdisciplinary approach to corn pest management research. For instance, the relationships between “semiochemicals,” “pests,” “formulation,” and “corn” demonstrate the multifaceted strategies that researchers are exploring to tackle pest problems. By studying different aspects of pest behavior, crop biology, and chemical formulations, researchers aim to develop biopesticides that are not only effective but also environmentally sustainable. The links between keywords suggest that researchers collaborate across various disciplines, combining insights from microbiology, genetics, and environmental science to innovate in biopesticides for corn.

**Fig. 8:**
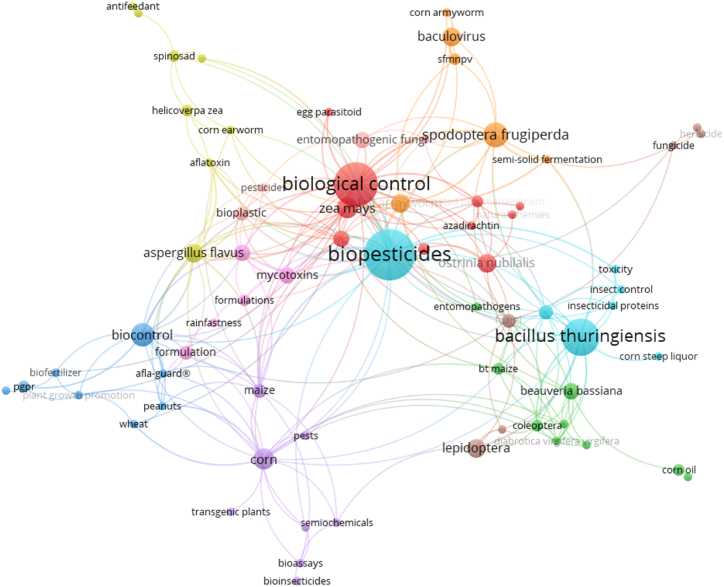
Co-occurrence of authors' keywords: minimum keywords threshold of 2 and 236 link.

In conclusion, the co-occurrence of authors' keywords in Fig. 8 highlights several dominant themes in biopesticides research for corn protection. These include the use of microbial and fungal biocontrol agents, strategies for controlling major pests like the fall armyworm, the development of effective biopesticide formulations, and the reduction of mycotoxin contamination in corn. The frequent appearance of keywords related to formulation challenges, transgenic plants, and pest-specific biopesticide strategies underscores the complexity and interdisciplinary nature of this field. The interconnectedness of these keywords reflects the broad scope of current research efforts aimed at enhancing sustainable corn production through the development and application of biopesticides.

### 3.9 Network view map generated by the VOSviewer

This section presents a network view map generated by the VOSviewer software, providing a visual representation of the bibliographic data analyzed in the context of biopesticides for corn protection. The VOSviewer tool enables the visualization of complex networks derived from bibliographic data, facilitating the exploration of relationships and patterns within the literature.

The network view map provides an intuitive visualization of the connections between various elements, such as authors, keywords, or terms, based on their co-occurrence or co-citation patterns. This approach reveals insights into underlying structures and thematic clusters within the body of literature on biopesticides for corn protection. This visual representation offers valuable insights into the key themes, trends, and relationships in the scholarly discourse surrounding biopesticides for corn protection. The exploration of the network view map generated by VOSviewer gives a deeper understanding of the interconnectedness and knowledge landscape within this field, paving the way for further analysis and interpretation.

**Fig. 9:**
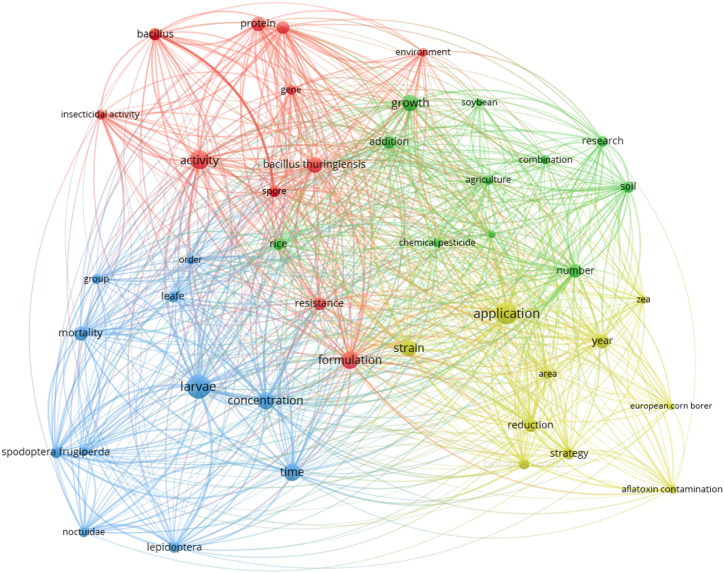
Network view map generated by the VOSviewer.

Fig. 9 presents a detailed and comprehensive analysis of recurring phrases within the research on biopesticides for corn protection. Larger circles on the map represent the most frequently mentioned phrases across the analyzed publications, indicating their significance in the research discourse. The key themes identified include “Application,” “Formulation,” “Activity,” and “Resistance.” These recurring phrases are essential elements in the study of biopesticides, highlighting the major focus areas for researchers. The phrases have been organized into five distinct clusters, each colored differently to visually distinguish the thematic groupings, offering an in-depth look at the research dynamics in the field.

The green-colored cluster draws attention to the combination of biopesticides and the various conditions and techniques associated with their use. This cluster emphasizes the practical deployment of biopesticides in agricultural systems, focusing on how different biopesticide formulations are applied in varying environmental conditions. It explores factors such as environmental influences (rain, temperature, humidity) and application techniques that affect the efficacy of biopesticides. The use of these biopesticides is shown to be highly versatile, with researchers investigating multiple approaches to enhance their performance under diverse agricultural conditions. The recurring focus on “combination” suggests that integrating biopesticides with other pest management strategies or combining different biopesticide agents is a joint research theme to improve overall pest control.

In contrast, the blue-colored cluster emphasizes investigating the concentration of biopesticides and their impact on pest mortality. This cluster underscores the importance of determining the optimal dosage and concentration levels to ensure effective pest control. The research in this cluster focuses heavily on understanding the quantitative aspects of biopesticide use—how much biopesticide is needed to effectively reduce pest populations without causing unintended environmental harm. The concentration of biopesticides is crucial because a dose that is too low might lead to inefficacy. At the same time, a dose that is too high could result in unwanted side effects, including non-target species harm or environmental degradation. This cluster highlights the need for precise formulation and careful monitoring of biopesticide application in agricultural fields.

The yellow-colored cluster is centered on the practical application of biopesticides in corn protection. This cluster delves into the methods and practices used in applying biopesticides on corn fields, addressing the timing, dosage, and delivery techniques that maximize their effectiveness. Researchers in this cluster examine various application technologies, such as spray systems, seed treatments, and soil applications, to determine the best methods for incorporating biopesticides into existing agricultural practices. The emphasis here is on ensuring that biopesticides are applied in a manner that provides consistent, reliable results while being practical and cost-effective for farmers. The discussions on application techniques also often explore innovations in precision agriculture, where biopesticides are applied more efficiently using advanced technologies.

The red-colored cluster is primarily associated with the microbial activity of biopesticides, highlighting the role of microorganisms in pest management. This cluster delves deep into the biological mechanisms through which microbial agents, such as *Bacillus thuringiensis*, *Beauveria bassiana*, and other entomopathogenic fungi, interact with pests. These microorganisms are critical components of many biopesticides because they infect and kill pests through biological processes, such as producing toxins or disrupting their metabolic processes. Research in this cluster focuses on understanding how these microbial agents work at a biological level, their specificity to particular pests, and how they can be optimized to control pest populations. Additionally, this cluster investigates the interactions between biopesticide microorganisms and their environment, ensuring that the biological control methods are practical, sustainable, and safe for non-target organisms.

Overall, this thematic clustering in Fig. 9 provides a nuanced understanding of the significant focus areas in biopesticide research for corn protection. By categorizing the recurring phrases into clusters, the figure offers insights into the priorities of researchers in this field: how to effectively apply biopesticides, optimize their formulation and concentration, understand their biological activity, and mitigate resistance. Each cluster represents a specific domain of expertise, and their interconnectedness shows the complexity and interdisciplinarity required to develop effective biopesticides for sustainable agriculture. The detailed analysis of these clusters contributes to a broader understanding of the research landscape, helping guide future studies and innovations in using biopesticides to protect corn crops from pests.

## 4 Recommendation for future research

Several future research directions emerge based on the co-occurrence of keywords in biopesticides for corn protection. One key area is the integration of biopesticides with other pest management strategies. The prominence of terms like “biological control” and “biopesticides” alongside specific pests such as *Spodoptera frugiperda* suggests that future research should explore the combination of biopesticides with crop rotation, biological control agents, and semiochemicals. This integrated pest management (IPM) approach could enhance the overall effectiveness of pest control while reducing dependency on chemical pesticides. Additionally, advancements in formulation technologies, highlighted by keywords such as “formulation” and “nanoparticles,” are crucial. Future studies should focus on improving biopesticide delivery systems, including nanoformulations and encapsulation techniques, to increase their stability and effectiveness under diverse environmental conditions.

Another primary research focus is addressing pest resistance development, as indicated by keywords like “resistance” and “insect control.” The growing resistance to Bt-based products calls for continuous monitoring and new strategies, such as rotating biopesticides or exploring novel methods like RNA interference (RNAi) and CRISPR. Expanding the use of microbial biopesticides, particularly *Bacillus thuringiensis* and *Beauveria bassiana*, is also critical. Research should explore new microbial strains and their interactions with pests and the environment, ensuring that microbial agents are sustainable and effective in diverse agricultural settings.

Climate resilience is another key priority, as evidenced by keywords related to environmental conditions like “rainfastness.” Developing biopesticides that can perform under extreme weather conditions, such as high temperatures or variable moisture levels, will ensure their effectiveness across different regions and climate scenarios. Plant-based biopesticides, such as those containing natural compounds like “azadirachtin” and “linalool,” are also gaining attention. Future research should focus on discovering novel plant-derived bioactive compounds and characterizing their efficacy and environmental safety.

Biotechnological approaches, particularly those involving gene editing and RNAi, will also play an essential role in the future of biopesticides, as suggested by keywords like “Bt maize” and “transgenic plants.” Research should focus on advancing these technologies to create more targeted, environmentally friendly pest control solutions. Alongside these innovations, long-term ecological and economic impact assessments will be vital. Understanding the broader effects of biopesticide use on biodiversity, soil health, and non-target species, as well as their economic feasibility, will help guide sustainable adoption.

Finally, there is a need to enhance the adoption of biopesticides through education and policy support. Despite their benefits, biopesticide use remains limited in some regions. Research should investigate barriers to adoption, such as cost, availability, and knowledge gaps, and propose strategies to overcome these challenges. Programs focused on educating farmers and supporting the commercialization of new biopesticide technologies will be essential for promoting widespread use. These future directions emphasize the need for continued innovation, sustainability, and practical application of biopesticides in corn crop protection.

## 5 Summary

In summary, future research in biopesticides for corn protection should prioritize enhancing formulation techniques to improve efficacy and stability, particularly under variable environmental conditions. Additionally, investigations into integrated pest management (IPM) strategies, combining biopesticides with cultural practices, biological control agents, and host plant resistance, can lead to more holistic and sustainable approaches to corn pest management. Comprehensive assessments of the ecological impact of biopesticides are essential, focusing on non-target organisms, soil health, and overall ecosystem dynamics to ensure compatibility with agroecological principles and sustainable agricultural practices. By integrating ecological monitoring approaches and promoting evidence-based decision-making, researchers can optimize biopesticide use to minimize environmental risks, promote ecosystem resilience, and foster sustainable crop production in corn agroecosystems.

## Declaration of generative AI and AI-assisted technologies in the writing process

During the preparation of this work the author(s) used Quilbott in order to paraphrase the language and check the language grammar. After using this tool/service, the author(s) reviewed and edited the content as needed and take(s) full responsibility for the content of the publication.

## CRediT authorship contribution statement

**Amik Krismawati:** Writing – review & editing, Writing – original draft, Validation, Data curation, Conceptualization. **Yustisia Yustisia:** Writing – review & editing, Writing – original draft, Data curation, Conceptualization. **Zainal Arifin:** Writing – review & editing, Writing – original draft, Data curation, Conceptualization. **Titik Purbiati:** Writing – review & editing, Writing – original draft, Conceptualization. **Diding Rachmawati:** Writing – review & editing, Writing – original draft, Data curation, Conceptualization. **Evy Latifah:** Writing – review & editing, Writing – original draft, Data curation, Conceptualization. **Nicky Rahmana Putra:** Writing – review & editing, Writing – original draft, Validation, Supervision, Data curation, Conceptualization. **Irianto Irianto:** Writing – review & editing, Writing – original draft, Visualization, Validation, Funding acquisition, Data curation, Conceptualization. **Lailatul Qomariyah:** Writing – review & editing, Writing – original draft, Supervision, Data curation, Conceptualization.

## Data availability

The data that support the findings of this study are available from the corresponding author, upon reasonable request.

## Declaration

We confirm that this manuscript is not under consideration elsewhere and that all authors have consented to its submission.

## Declaration of competing interest

The authors declare that they have no known competing financial interests or personal relationships that could have appeared to influence the work reported in this paper.
